# Prospective surveillance for influenza A virus in Chinese swine farms

**DOI:** 10.1038/s41426-018-0086-1

**Published:** 2018-05-16

**Authors:** Benjamin D. Anderson, Mai-Juan Ma, Guo-Lin Wang, Zhen-Qiang Bi, Bing Lu, Xian-Jun Wang, Chuang-Xin Wang, Shan-Hui Chen, Yan-Hua Qian, Shao-Xia Song, Min Li, Teng Zhao, Meng-Na Wu, Laura K. Borkenhagen, Wu-Chun Cao, Gregory C. Gray

**Affiliations:** 1 Division of Infectious Diseases, School of Medicine, Global Health Institute, Duke University, Durham, NC 27710 USA; 2 Global Health Research Center, Duke-Kunshan University, 215316 Kunshan, China; 3 State Key Laboratory of Pathogen and Biosecurity, Beijing Institute of Microbiology and Epidemiology, 100071 Beijing, China; 4 Shandong Provincial Center for Disease Control and Prevention, 250013 Jinan, China; 5 Shandong Provincial Key Laboratory of Disease Control and Prevention, 250013 Jinan, China; 6 Wuxi Center for Disease Control and Prevention, 214023 Wuxi, China; 7 Licheng District Center for Disease Control and Prevention, 250199 Jinan, China; 8 Program in Emerging Infectious Diseases, Duke-NUS Medical School, Singapore, 169857 Singapore

## Abstract

Pork production in China is rapidly increasing and swine production operations are expanding in size and number. However, the biosecurity measures necessary to prevent swine disease transmission, particularly influenza A viruses (IAV) that can be zoonotic, are often inadequate. Despite this risk, few studies have attempted to comprehensively study IAV ecology in swine production settings. Here, we present environmental and animal sampling data collected in the first year of an ongoing five-year prospective epidemiological study to assess IAV ecology as it relates to swine workers, their pigs, and the farm environment. From March 2015 to February 2016, we collected 396 each of environmental swab, water, bioaerosol, and fecal/slurry samples, as well as 3300 pig oral secretion samples from six farms in China. The specimens were tested with molecular assays for IAV. Of these, 46 (11.6%) environmental swab, 235 (7.1%) pig oral secretion, 23 (5.8%) water, 20 (5.1%) bioaerosol, and 19 (4.8%) fecal/slurry specimens were positive for influenza A by qRT-PCR. Risk factors for IAV detection among collected samples were identified using bivariate logistic regression. Overall, these first year data suggest that IAV is quite ubiquitous in the swine production environment and demonstrate an association between the different types of environmental sampling used. Given the mounting evidence that some of these viruses freely move between pigs and swine workers, and that mixing of these viruses can yield progeny viruses with pandemic potential, it seems imperative that routine surveillance for novel IAVs be conducted in commercial swine farms.

## Introduction

The United States Department of Agriculture (USDA) predicted that 2017 would be a record year for worldwide pork production, increasing approximately 1% from 2016 and 0.3% greater than the current record set in 2014^1^. Much of this production is due to the pork industry in China, which accounted for nearly half of the pork produced globally in 2017. Pork production in China has increased two-fold during the last 30 years^1,2^. Worldwide swine production operations are increasing in size and number and pork production overall is subsequently becoming more efficient. However, the biosecurity measures necessary to prevent the spread of highly infectious diseases, such as porcine reproductive and respiratory syndrome and foot and mouth disease, as well as emerging zoonotic diseases, often remains inadequate^3–5^.

One group of zoonotic pathogens of major concern are influenza A viruses (IAV), of which subtypes H1 and H3 are known to circulate in both pigs and humans^6,7^. Subtype H1N1 has been endemic among pigs since at least 1918, however, more recently the lineage has been associated with a rising number of swine-origin IAVs among humans^6,7^. It seems likely that the 2009 H1N1 pandemic virus originated in pigs in Mexico^8^ causing an estimated 151,700 human deaths^9^. Recently, a progeny of that pandemic virus, an H3N2 variant, has been identified as a potential pandemic threat due to increased incidence since 2011^10^. These viruses occassionally infect humans, especially those who are exposed to pigs in agricultural fairs^11^. The 2009 pandemic and the recent outbreaks of variant influenza A viruses illustrate the importance of new and greater surveillance at the interface of animals and humans.

To understand IAV ecology as it relates to swine production, we are conducting a five-year prospective epidemiologic study assessing swine workers, their pigs, and the farm environment^12^. Here, we describe in detail the results and analysis of the environmental and animal sampling data collected in the first year of the study.

## Methods

### Study design

This study was approved by the institutional review boards of Duke University (Pro00056116) and the Academy of Military Medical Sciences (no number given). Institutional Animal Care and Use Committee approvals were also granted by Duke University (A187-14-08) and the Academy of Military Medical Sciences (AMMS-20-14-009). Six Chinese pig farms (three each in Jiangsu and Shandong Provinces) were visited monthly to collect four types of environmental samples: 1) a convenience sample of six environmental swab specimens from barn areas where workers and pigs were likely to share contact; 2) a convenience sample of six pen-side fresh or swine waste water samples; 3) a convenience sample of six pig fecal/slurry samples; and 4) a convenience sample of six, 30-minute aerosol samples. Study personnel also collected a convenience sample of 50 pig oral secretion samples during each monthly farm visit.

### Data collection

At the beginning of each farm visit, the farm owner or manager was asked to complete a questionnaire assessing various descriptive details of their facility, including the number of animals on site and the health status of their swine herds. Study personnel also measured the temperature outside and inside of sampled barns, the relative humidity, and recorded the current weather conditions. Data captured for each environmental and pig oral secretion collection included the sampling location, the age, and type of pigs in each pen in closest proximity.

### Sample collection

Environmental and pig oral secretion sampling were conducted concomitantly, with the goal of collecting all samples within the same area of each sampled barn. Additionally, sampling areas were selected such that each type of pig (unweaned pigs, weaned pigs, growers, finishers, sows, and boars) were included. These criteria were applied consistently across farms.

#### Bioaerosol

Bioaerosol sampling was conducted using BioSamplers (SKC, Inc., Eighty Four, PA; catalog number 225-9595) operated with 220-volt SKC BioLite sampling pumps (SKC Inc., Eighty Four, PA; catalog number 228-9610), as previously described^13,14^. Briefly, the sampler was connected to in-line vapor traps (SKC Inc., Eighty Four, PA; catalog number 225-22-01) to protect the pumps against moisture and pumps were allowed to run for 5 min before sampling as a warm-up. The SKC BioSampler was filled with 15 ml of sterile phosphate buffered saline (PBS) with 0.5% (w/v) bovine serum albumin fraction V (BSA) powder and placed 1–1.5 m above the ground. Each sampling pump was operated at a flow rate of 8 /l/min for 30 minutes allowing for the sampling of approximately 240 liters of air per site. At the end of the sampling period, the pump was shut off, the BioSampler disconnected, and sample media aseptically transferred from the SKC BioSampler collection vessel into a sterile 15 ml conical tube. All samplers were disinfected by autoclave at the end of each sampling day.

#### Environmental swab

Environmental swab specimens were collected and preserved using a commercial viral swab kit with viral transport media in a transport tube (MT0301, Yocon, Beijing, China). Swab samples were collected by swabbing surfaces that were likely to be touched by both humans and pigs, including the pigpen gates and walls.

#### Water

Using aseptic technique and sterile collection bottles, fresh water was collected in 500 ml volumes from water dispensers located inside the pigpens and swine waste water was collected in 50–200 ml volumes from the open waste water drains located inside each sampled barn.

#### Fecal slurry

Fecal material (1 g) or slurry (~1 ml) samples were collected from within the pigpen or from the waste catchment drains.

#### Pig oral secretion

Pig oral secretion samples were collected using a hanging rope method which has been previously described^15–17^. Briefly, 3-strand braided unbleached 100% cotton ropes, with 5/8” diameter, were pre-soaked with a 5% sterile glucose solution and placed in the pig pens. Ropes were attached to a rod or pole and placed 40 cm above the ground for 20 to 30 mins during which time the pigs would chew on the rope. At the conclusion of the sampling, and using aseptic technique, oral fluids were manually expressed from the rope into a Labplas Twirl’ EM Sterile Sampling Bag (Cat. No. EPR-4590, Labplas, Inc., Canada).

### Sample handling, storage, and shipment

All samples were placed on wet ice immediately after collection and transported to the nearest collaborating CDC laboratory where they were aliquoted into two equal parts and preserved at −80 °C. All specimens were labeled with farm ID, date of sample collection, and sampling site within the farm. Samples were periodically transported on dry ice to the Beijing Institute of Microbiology and Epidemiology-Academy of Military Medical Sciences, Beijing, China (IME-AMMS), where processing and laboratory testing were conducted.

### Sample processing

Fecal/slurry samples were diluted in 4 ml of Minimum Essential Medium (Invitrogen Grand Island, NY) containing 10% glycerin, penicillin (2000 U/ml), gentamicin (250 mg/ml), polymixin B (2000 U/ml), and nystatin (500 U/ml) to obtain a final 25% suspension (w/v). For fresh water samples, a final concentration of 0.05% suspension of formaldehyde-fixed turkey erythrocytes in PBS (10% concentration) was added to the water (500 ml–2 l). Samples were then mixed on a shaker at 10 min intervals for one hour on wet ice, after which they were aliquoted into sterile 250 ml centrifuge bottles and centrifuged for 5 min at 5000 r.p.m. in a 4 °C pre-chilled rotor chamber. The supernatant was discarded, and 2 ml of liquid was left in each bottle. For collected pig waste water samples, 50–200 ml samples were mixed with 9× volume of sterile water and adapted to the fresh water procedure.

### Laboratory analyses

The viral RNA from each environmental and pig oral secretion specimen was extracted using MiniBEST viral RNA/DNA Extraction Kits (Cat. No. 9766, TaKaRa, Dalian, China). Positive and negative controls were used during each extraction to validate the extraction procedure and reagent integrity. The viral RNA of each sample was screened for influenza A virus by a qRT-PCR assay targeting the influenza matrix genome segment^18^ using a one-step RT-PCR kit (Cat. No. 56046, TaKaRa, Dalian, China) on an Applied Biosystems 7500 real-time PCR platform (Life Technologies, NY, USA). All qRT-PCR runs had a negative template control, and the corresponding primer set viral positive template control. Real-time RT-PCR samples with a Ct value less than or equal to 38 were considered positive.

Influenza A virus qRT-PCR positive specimens with Ct values ≤30 were subsequently inoculated into 9–11 day old embryonated chicken eggs or onto MDCK cells and incubated at 35 °C or 38 °C and observed for up to 7 days for attempts at virus isolation. Allantoic fluid from eggs or MDCK cell supernatant were screened with qRT-PCR. For the specimens that were culture-positive, the entire viral genomes were then amplified using a pair of universal primers^19^ and a one-step conventional RT-PCR Kit (Cat. No. 055A, TaKaRa, Dalian, China). Next, RT-PCR amplicons were purified with a MinElute PCR Purification Kit (Cat. No. 28004, QIAGEN) and then sequenced on an Ion Torrent Personal Genome Machine (PGM, Life Technologies, South San Francisco, CA).

For the culture-negative specimens with Ct value ≤30, attempts were made to directly amplify viral RNA from the original specimen and to sequence the whole viral genome using the Ion Torrent sequencing mentioned above. Finally, for the remaining unidentified qRT-PCR-positive specimens with Ct values ≤36 attempts were made to characterize the viruses through the use of HA, NA, and M gene universal primers^20,21^ with a one-step RT-PCR Kit (Cat. No. 055 A, TaKaRa, Dalian, China). Amplicons were sequenced using standard techniques on a 3730 xl DNA Genetic Analyzer (Applied BioSystems, Inc., Foster City, CA) using the Big Dye Terminator Kit v3.1 (Applied BioSystems, Inc., Foster City, CA).

### Data analysis

Bivariate *χ*2 tests of independence or Fisher exact tests were used to examine the strength of association for potential risk factors for IAV among environmental and pig oral secretion samples. Variables determined by bivariate analyses to be statistically associated with positivity (*p* < 0.10) were then analyzed using bivariate logistic regression to obtain odds ratios and 95% confidence intervals. Correlation statistics were calculated using Cohen’s Kappa to compare agreement between the different types of environmental sampling. Statistical analysis was performed using STATA 14.1 (StataCorp, College Station, TX).

## Results

Six farms were sampled between March 2015 and February 2016. Farm characteristics have been previously described, but in brief they varied in size and biosecurity^12^. A total of 396 each of environmental swab, water, bioaerosol, and fecal/slurry specimens and 3300 pig oral secretion specimens were collected and tested. Of these, 46 (11.6%) environmental swabs, 235 (7.1%) pig oral secretions, 23 (5.8%) water, 20 (5.1%) bioaerosol, and 19 (4.8%) fecal/slurry specimens were positive for influenza A by qRT-PCR. In total, 28 samples yielded sequence data identifying swine H1N1 (*n* = 11), swine H3N2 (*n* = 2), A(H1N1)pdm09-like virus (*n* = 5), swine subtype H1 (*n* = 3), swine subtype N1 (*n* = 6), and A(H1N1)pdm09-like virus N1 (*n* = 1) viruses. As has been previously reported, the A(H1N1)pdm09-like viruses detected in the swine were nearly genetically identical to viruses detected in swine workers also studied at the same farms^12^.

**Fig. 1:**
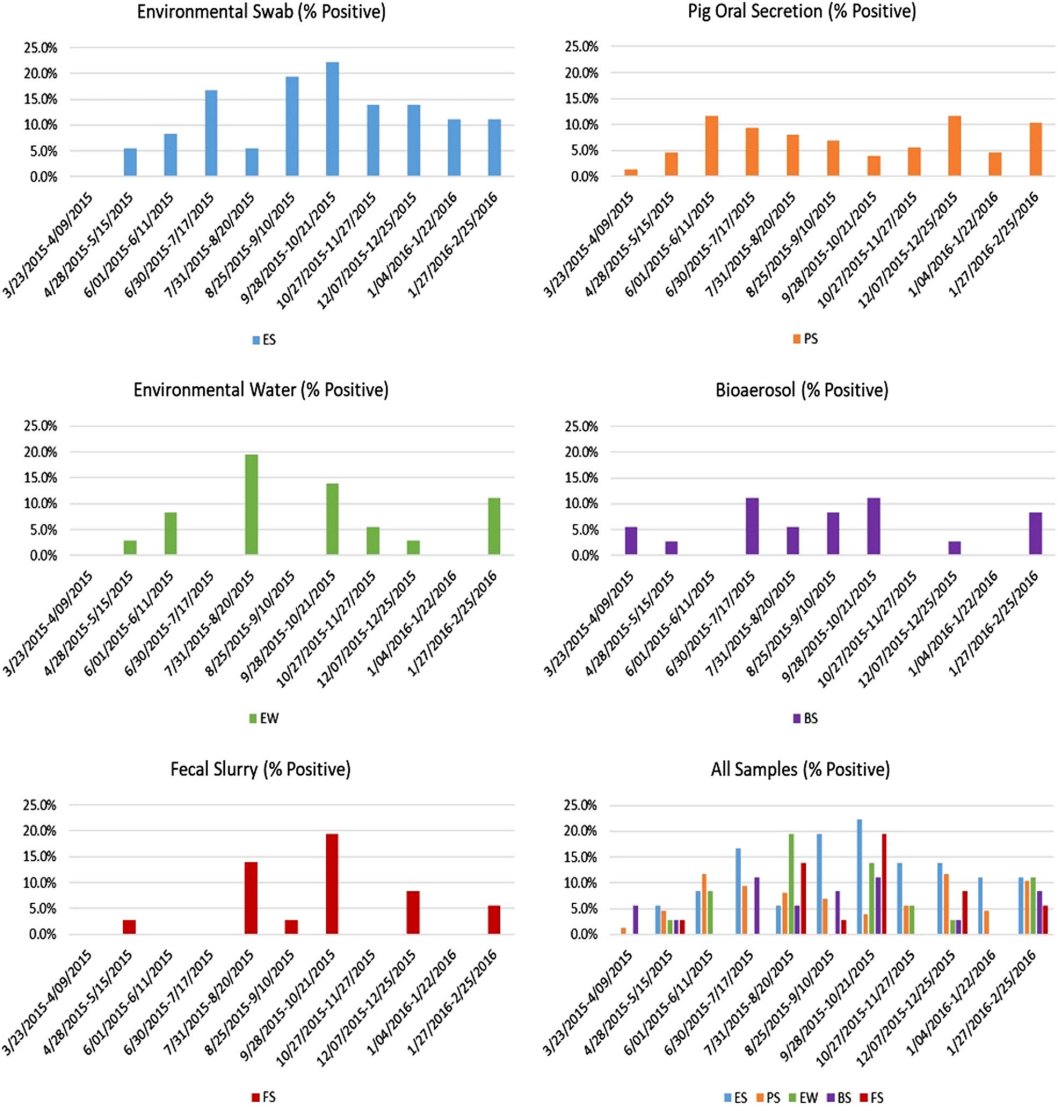
Influenza A virus molecular prevalence by sample type collected monthly from six Chinese swine farms (three in Shandong and three in Jiangsu Province) between 23 March 2015 and 25 February 2016 . ES environmental swab, PS pig oral secretion, EW environmental water, BS bioaerosol, FS fecal slurry

**Fig. 2:**
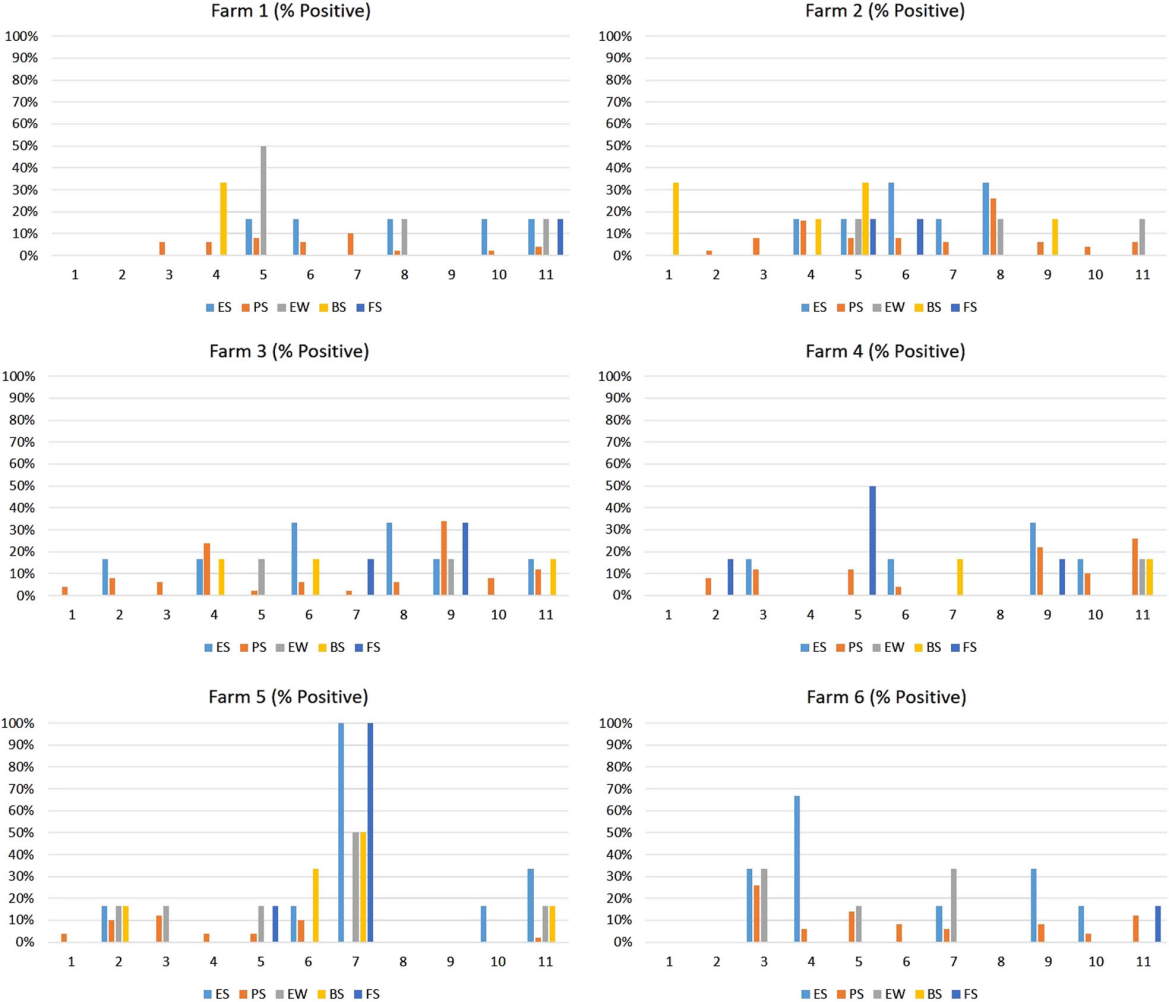
Influenza A virus molecular prevalence by farm collected monthly from six Chinese swine farms (three in Shandong and three in Jiangsu Province) between 23 March 2015 and 25 February 2016 . ES environmental swab, PS pig oral secretion, EW environmental water, BS bioaerosol, FS fecal slurry

The distribution of influenza positives during the sampling period appeared to be bimodal for environmental swab, pig oral secretion, bioaerosol, and environmental water specimens, with peaks occurring during the warmer summer months (June-August) and the cooler fall/winter months (September-February) (Fig. 1). Only a single peak of influenza positivity was observed during the summer for fecal/slurry sampling. When the distribution of influenza A positives was stratified by farm (Fig. 2), the distribution of influenza positives by sample type varied. Notably, farm 5 had a spike in influenza A positivity in environmental swab, environmental water, bioaerosol, and fecal slurry samples during September 2015.

### Risk factor analysis

Significant risk factors for influenza A positivity in environmental swab samples (Table 1) included outside temperatures 5.0 °C–13.9 °C (OR = 3.06, 95% C.I. 1.04–8.98) and outside temperatures 14.0–23.9 °C (OR = 3.44, 95% C.I. 1.08–10.95). Seasonality was also a significant predictor, with samples collected in the summer (OR = 3.32, 95% C.I. 1.16–9.50) and fall (OR = 4.12; 95% C.I. 1.47–11.54), using spring as the reference, having a higher odds of influenza A positivity. There was strong association of influenza A positivity in environmental swab sampling with concomitantly collected environmental water (OR = 3.75, 95% C.I. 1.45–9.67), fecal/slurry (OR = 5.06, 95% C.I. 1.88–13.60), and bioaerosol (OR = 2.72, 95% C.I. 0.94–7.88) samples.

**Table 1:** Unadjusted odds ratios for risk factors associated with influenza A molecular positivity among 396 environmental surface swab samples collected from six Chinese pig farms (three farms from Shandong and three farms from Jiangsu Province) between March 2015 and February 2016

	Environmental surface swab influenza A positive		
Risk factor	Total *N*	No. (%)	Unadjusted OR (95% CI)
Temperature outside of sampled barn (°C)			
−5.0–4.9	72	6 (8.3)	1.0 (0.29–3.45)
5.0–13.9	48	8 (16.7)	**3.06 (1.04–8.98)**
14.0–23.9	42	10 (23.8)	**3.44 (1.08–10.95)**
24.0–29.9	120	15 (12.5)	1.57 (0.54-4.55)
≥30.0	60	5 (8.3)	Ref.
Season sample was collected^a^			
Winter	72	8 (11.1)	2.56 (0.81-8.21)
Fall	108	18 (16.7)	**4.12 (1.47–11.54)**
Summer	108	15 (13.9)	**3.32 (1.16–9.50)**
Spring	108	5 (4.6)	Ref.
Environmental water sample influenza A virus positivity			
Positive	23	7 (30.4)	**3.75 (1.45–9.67)**
Negative	373	39 (10.5)	Ref.
Fecal slurry sample influenza A virus positivity			
Positive	19	7 (36.8)	**5.06 (1.88–13.60)**
Negative	377	39 (10.3)	Ref.
Bioaerosol sampling influenza A virus positivity			
Positive	20	5 (25.0)	2.72 (0.94–7.88)
Negative	376	41 (10.9)	Ref.

CI confidence interval

ORs significant with a *p*-value < 0.05 are indicated in bold text

^a^Seasonality: spring (March 23, 2015 through June 20, 2015), summer (June 21, 2015 through Sept. 22, 2015), fall (Sept. 23, 2015 through Dec. 20, 2015), winter (Dec. 21, 2015 through Feb. 25, 2016)

Using pig oral secretion influenza A positivity as the outcome (Table 2), significant predictors included the number of pigs onsite, number of non-swine animals onsite (poultry and/or dogs), the temperature outside of the sampled barn, humidity, type of pigs, and whether pigs showed clinical signs of illness in the past two weeks. Age of pigs was also a statistically significant predictor, with the youngest pigs aged 1–10 days old having the greatest odds of IAV positivity (OR = 4.13, 95% C.I. 2.23–7.63). Using environmental water influenza A positivity as the outcome (Table 3), temperature outside was a significant predictor. Using bioaerosol influenza A positivity as the outcome (Table 4), pigs showing signs of clinical illness in the last two weeks, disposal method for ill or dead animals, and the total number of pigs on site were significant predictors.

**Table 2:** Unadjusted odds ratios for risk factors associated with influenza A molecular positivity among 3300 pig oral secretion samples collected from six Chinese pig farms (three farms from Shandong and three farms from Jiangsu Province) between March 2015 and February 2016

	Pig oral secretion influenza A positive		
Risk factor	Total N	No. (%)	Unadjusted OR (95% CI)
Total number of pigs onsite			
≥3000	250	10 (4.0)	0.78 (0.37–1.62)
2000–2999	700	51 (7.3)	1.47 (0.91–2.36)
1000–1999	1350	122 (9.0)	**1.85 (1.21–2.83)**
<1000	550	28 (5.1)	Ref.
Total number of non-swine animals onsite*			
≥30	550	28 (5.1)	0.69 (0.44–1.10)
20–29	850	87 (10.2)	**1.47 (1.05–2.08)**
10–19	600	35 (5.8)	0.80 (0.52–1.23)
<10	850	61 (7.2)	Ref.
Temperature outside of sampled barn (°C)			
−5.0–4.9	600	25 (4.2)	**0.40 (0.24–0.66)**
5.0–13.9	400	39 (9.8)	0.99 (0.64–1.55)
14.0–23.9	350	30 (8.6)	0.86 (0.54–1.39)
24.0–29.9	1000	68 (6.8)	**0.67 (0.46-0.99)**
≥30.0	500	49 (9.8)	Ref.
Humidity quintiles (%)			
35.0–49.0 (Q1)	650	44 (6.8)	**0.62 (0.41–0.93)**
50.0–62.0 (Q2)	500	35 (7.0)	**0.64 (0.41–0.99)**
63.0–75.0 (Q3)	650	44 (6.8)	**0.62 (0.41–0.93)**
76.0–79.0 (Q4)	500	30 (6.0)	**0.54 (0.34–0.86)**
80.0–94.0 (Q5)	550	58 (10.6)	Ref.
Type of pigs near where samples were collected			
Sow/weaning pig	10	1 (10.0)	1.64 (0.19–14.43)
Sow	632	16 (2.5)	**0.38 (0.17–0.89)**
Pregnant sow	20	3 (15.0)	2.61 (0.64–10.58)
Weaning pig	5	1 (20.0)	3.69 (0.37–36.59)
Production pig	2491	205 (8.2)	1.33 (0.66–2.64)
Boar	142	9 (6.3)	Ref.
Pigs showed signs of illness in past 2 weeks (14 days)			
Yes	55	10 (18.2)	**2.98 (1.48–6.0)**
No	3245	225 (6.9)	Ref.
Age of pigs in weeks near where sample were collected (quintiles)			
1.0–10.0	782	91 (11.6)	**4.13 (2.23–7.63)**
11.0–16.0	788	54 (6.9)	**2.31 (1.22–4.36)**
17.0–20.0	439	29 (6.6)	**2.22 (1.11–4.41)**
21.0–78.0	903	49 (5.4)	1.80 (0.95–3.42)
79.0–260.0	388	12 (3.1)	Ref.

CI confidence interval

ORs significant with a *p*-value < 0.05 are indicated in bold text

*Non-swine animals include poultry and dogs

**Table 3:** Unadjusted odds ratios for risk factors associated with influenza A molecular positivity among 396 environmental water samples collected from six Chinese pig farms (three farms from Shandong and three farms from Jiangsu Province) between March 2015 and February 2016

	Environmental water influenza A positive		
Risk factor	Total N	No. (%)	Unadjusted OR (95% CI)
Temperature outside of sampled barn (°C)			
−5.0–4.9	72	2 (2.8)	**0.16 (0.03–0.78)**
5.0–13.9	48	2 (4.2)	0.25 (0.05–1.20)
14.0–23.9	42	5 (11.9)	1.27 (0.41–3.89)
24.0–29.9	120	3 (2.5)	**0.15 (0.04–0.56)**
≥30.0	60	9 (15.0)	Ref.
Fecal slurry sample influenza A virus positivity			
Positive	19	5 (26.3)	**7.12 (2.31–21.95)**
Negative	377	18 (4.8)	Ref.
Environmental surface swab sample influenza A virus positivity			
Positive	46	7 (15.2)	**3.75 (1.45–9.67)**
Negative	350	16 (4.6)	Ref.
Bioaerosol sampling influenza A virus positivity			
Positive	20	3 (15.0)	3.14 (0.85–11.61)
Negative	376	20 (5.3)	Ref.

CI confidence interval

ORs significant with a *p*-value < 0.05 are indicated in bold text

**Table 4:** Unadjusted odds ratios for risk factors associated with influenza A molecular positivity among 396 bioaerosol samples collected from six Chinese pig farms (three farms from Shandong and three farms from Jiangsu Province) between March 2015 and February 2016

	Bioaerosol influenza A positive		
Risk factor	Total N	No. (%)	Unadjusted OR (95% CI)
Pigs showed signs of illness in past 2 weeks (14 days)			
Yes	216	14 (6.5)	4.30 (0.96–19.23)
No	126	2 (1.6)	Ref.
Fate of ill or dead animals			
Buried or destroyed	48	6 (12.5)	**8.86 (1.72–45.57)**
Animal food	132	8 (6.1)	4.0 (0.83–19.21)
Sold to market	126	2 (1.6)	Ref.
Total number of pigs on site			
≥3000	36	6 (16.7)	4.2 (0.98–17.95)
2000–2999	84	4 (4.8)	1.05 (0.23–4.86)
1000–1999	156	3 (1.9)	0.41 (0.08–2.10)
<1000	66	3 (4.6)	Ref.
Environmental water sample influenza A virus positivity			
Positive	46	5 (10.9)	3.14 (0.85–11.61)
Negative	350	15 (4.3)	Ref.
Fecal slurry sample influenza A virus positivity			
Positive	19	3 (15.8)	**3.97 (1.05–14.95)**
Negative	377	17 (4.5)	Ref.
Environmental surface swab sample influenza A virus positivity			
Positive	46	5 (10.9)	2.72 (0.94–7.88)
Negative	350	15 (4.3)	Ref.

CI confidence interval

ORs significant with a *p*-value < 0.05 are indicated in bold text

Last, using fecal/slurry influenza A positivity as the outcome (Table 5), the total number of pigs onsite, the total number of non-swine animals onsite, temperature outside the barn, season, and age of pigs were significant predictors for IAV positivity in samples. Specifically, in contrast to the findings for the pig oral secretion samples that showed the youngest pigs to have the highest odds of influenza A detection, it was the oldest pigs (105-208 days old), near where fecal slurry samples were collected, that had the highest odds of IAV positivity. The detailed output for the bivariate and logistic regression analysis results are included as Supplementary Information.

**Table 5:** Unadjusted odds ratios for risk factors associated with influenza A molecular positivity among 396 fecal/slurry samples collected from six Chinese pig farms (three farms from Shandong and three farms from Jiangsu Province) between March 2015 and February 2016

	Fecal/slurry influenza A positive		
Risk factor	Total N	No. (%)	Unadjusted OR (95% CI)
Total number of pigs on site			
≥3000	36	6 (16.7)	**13.0 (1.50–112.81)**
2000–2999	84	2 (2.4)	1.59 (0.14–17.87)
1000-1999	156	9 (5.8)	3.98 (0.49–32.06)
<1000	66	1 (1.5)	Ref.
Total number of non-swine animals onsite^a^			
≥30	60	10 (16.7)	**10.0 (2.11–47.38)**
20–29	102	3 (2.9)	1.52 (0.25–9.26)
10–19	78	3 (3.9)	2.0 (0.33–12.27)
<10	102	2 (2.0)	Ref.
Temperature outside of sampled barn (°C)			
−5.0–4.9	72	1 (1.4)	0.41 (0.04–4.62)
5.0–13.9	48	3 (6.3)	1.93 (0.31–12.07)
14.0–23.9	42	7 (16.7)	**5.8 (1.14–29.50)**
24.0–29.9	120	5 (4.2)	1.26 (0.24–6.70)
≥30.0	60	2 (3.3)	Ref.
Season sample was collected^b^			
Winter	72	2 (2.8)	3.06 (0.27–34.36)
Fall	108	10 (9.3)	**10.92 (1.37–86.86)**
Summer	108	6 (5.6)	6.29 (0.74–53.2)
Spring	108	1 (0.9)	Ref.
Age of pigs in weeks near where samples were collected (quintiles)			
105.0–208.0 (Q5)	62	8 (12.9)	**5.23 (1.33–20.53)**
53.0–104.0 (Q4)	91	1 (1.1)	0.39 (0.04–3.84)
21.0–52.0 (Q3)	51	5 (9.8)	3.84 (0.88–16.75)
13.0–20.0 (Q2)	83	2 (2.4)	0.87 (0.14–5.34)
1.0–12.0 (Q1)	109	3 (2.8)	Ref.
Environmental water sample influenza A virus positivity			
Positive	23	5 (21.7)	**3.75 (1.45–9.67)**
Negative	373	14 (3.8)	Ref.
Environmental surface swab sample influenza A virus positivity			
Positive	46	7 (15.2)	**5.06 (1.88–13.60)**
Negative	350	12 (3.4)	Ref.
Bioaerosol sampling influenza A virus positivity			
Positive	20	3 (15.0)	2.72 (0.94–7.89)
Negative	376	16 (4.3)	Ref.

CI confidence interval

1ORs significant with a *p*-value < 0.05 are indicated in bold text

^a^Non-swine animals include poultry and dogs

^b^Seasonality: spring (March 23, 2015 through June 20, 2015), summer (June 21, 2015 through Sept. 22, 2015), fall (Sept. 23, 2015 through Dec. 20, 2015), winter (Dec. 21, 2015 through Feb. 25, 2016)

As demonstrated by bivariate logistic regression results (Tables 1–5), there was a measureable association in IAV detections between the environmental swab, environmental water, fecal/slurry, and bioaerosol specimens. Correlation calculations demonstrated a slight agreement across all environmental sampling methods (*κ* = 0.1256), with the strongest agreement measured between environmental water and fecal/slurry samples (*κ* = 0.1985).

## Discussion

In this report, we present the environmental and animal sampling data from the first year of a large, complex prospective 5-year epidemiological study of swine influenza virus being conducted in Chinese swine farms. Our results revealed a steady detection of IAV by qRT-PCR over time, both in the pigs and in multiple types of environmental samples, across the six swine farms studied in the first year. Risk factor analyses revealed statistically significant associations between different environmental and animal-related covariates, such as season and age of pigs. These findings, paired with the previously published first year human data showing molecular and serological evidence of swine influenza infection^12^, paints a complex picture of IAV ecology within swine production settings and supports the high probability of transmission within and between the human, animal, and environmental domains.

There was a subtle bimodal distribution of IAV molecular detection in pig oral secretions, with peaks at roughly 12% in June and December/January. Molecular detections of IAV in fecal/slurry samples also revealed peaks corresponding with seasonal change. Dual peaks of influenza A prevalence during the summer and winter months have been previously observed among human populations in China^22–25^, and have been documented in pig farms^26,27^. Similarly, though less pronounced likely due to a more limited sample size, bimodal distributions of IAV positivity were also observed in the other types of environmental samples collected, including environmental swabs and bioaerosol samples.

Influenza A virus was detected at the greatest prevalence in environmental swab samples. This is likely attributable to a greater accumulation and persistence of virus on barn surfaces where pigs and people had frequent shared contact. In contrast, bioaerosol sampling yielded the lowest prevalence of IAV detection. It is likely that ventilation, temperature, and humidity within the facilities were less conducive to aerosolization, thus reducing detection with the bioaerosol samplers^14^. Though, there was an association found between IAV detection and pigs showing signs of clinical illness in the previous two weeks. This suggests that virus was more likely to be detected in the air when pigs were actively infected and shedding virus, which is consistent with previous findings^28^.

Regarding IAV prevalence across the farms by specimen type, there was a noticeable spike in influenza A detections in the swab, water, fecal/slurry, and air specimens collected from Farm 5 during the seventh sampling month (September 2015) (Fig. 2). Surprisingly, there were no influenza A positives detected in the pig oral secretion specimens collected at the same time. We posit that the swine that were sampled were no longer shedding virus at the time of collection, suggesting environmental persistence of detected virus or contamination from other non-sampled pigs who were shedding virus in the farm elsewhere.

When assessing the age of pigs near where sampling was conducted as a covariate, data showed that fecal/slurry samples from pigs in the highest age quintile (105.0–208.0 weeks) were associated with the greatest odds of influenza A positivity (Table 5). In contrast, the odds of IAV positivity were greatest in oral secretion samples collected from pigs in the lowest age quintile (1.0–10.0 weeks) (Table 2). Previous studies support the notion that younger pigs or piglets are more likely to shed IAV, which is consistent with our findings^29,30^. It is possible that IAV was shed from the older pigs and was accumulated in the fecal/slurry material. Given the agreement in influenza A positivity between the water and fecal/slurry material, it is also possible that IAV in the water was contaminating the fecal/slurry material.

There were some limitations worth noting. Virus isolation for this study was attempted and the results previously published^12^. This data showed virus isolation for the environmental samples to be sparse, making it difficult to know if our molecular detections represented viable IAV. Virus isolation was much more frequent among pig oral secretion samples. However, given the prospective nature of the study and the frequent molecular detections identified along with the isolation of virus from pig samples, we infer that viable IAV is being readily sustained in both the pigs and the environment.

Overall, our findings show IAV to be relatively ubiquitous across the different types of environmental and pig specimens collected from all six surveyed farms located in Jiangsu and Shandong Provinces in the first year of this prospective study. Our data also demonstrated an association between the different types of environmental specimens collected and IAV detection. Our comprehensive environmental specimen collection strategy, which includes air sampling technology and sampling of the pig oral secretion, aims to better describe IAV ecology in swine farms in China, which are complex ecological environments. Given the evidence that IAV in these farms is likely freely moving between the pigs and workers^12^, with potential to yield new viruses, it seems extremely important that routine surveillance for novel IAV generation be included in swine production farms.

## Electronic supplementary material

Supplementary Information
